# Is urinary Na/K ratio an independent indicator associated with current hypertension and RA disease activity or just an artifact? Comment on article by Minamino H et al

**DOI:** 10.1186/s13075-021-02571-2

**Published:** 2021-07-08

**Authors:** Reza Rastmanesh

**Affiliations:** 1 The Nutrition Society, London, UK; 2 Independent Researcher, Private Clinic, Tehran, 1981635555 Iran

I read with interest the article by Minamino et al. [1]. I would like to clarify some of the significant findings and suggest re-analysis needed to validate their core conclusion.

Authors have concluded that urinary Na/K ratio is an independent indicator associated with current RA disease activity.

First, physiologically, with regard to dietary sodium intake, it is worth to remind that some people are more sodium-responsive [2]. In a recent study in Japanese evacuees after the Great East Japan Earthquake investigating salt intake and risk of disaster hypertension, “there was an interaction between estimated sodium intake and disaster hypertension according to the presence or absence of high risk of salt-sensitive hypertension in the group without prevalent hypertension (P=0.03)”. Considering that participants in the study of Minamino et al. [1] were enrolled from a cohort database (KURAMA) in a non-random manner, this raises a concern whether subpopulations existed in their study population (e.g., sodium-responsive subjects) in which adaptation to sodium-induced hypercalciuria [3] or sodium-induced hypokalemia [4] could be compromised.

With regard to hypertension, it is known that in sodium-responsive individuals, KCl prevents the rise in blood pressure induced by dietary salt; however, with a low sodium intake, no significant effect on blood pressure is seen. It is also known that potassium itself reduces the rise in blood pressure caused by sodium chloride intake and reduces the increased sympathetic postural response which is seen in people on low-sodium diets [3].

Minamino et al. [1] have estimated daily salt intake using Tanaka’s formula; however, there was no report on dietary potassium intake or an estimation of K intake.

Urinary potassium was initially suggested to be reliable for use as a recovery biomarker in dietary studies [5]; however, precautions should be taken into account, as these findings are not replicated in an outpatient setting. Furthermore, in subsequent replication study [6], researchers concluded that due to measurement error in one or both estimates from dietary and urine measures, correlations between dietary and urinary sodium or potassium is very low [6]. In Japanese population, this may be even more pronounced because of significant difference in terms of dietary sodium and potassium intake among people [7].

This raises another issue since relation between blood pressure and dietary salt can be modulated by other dietary factors, such as potassium, where potassium intake can blunt the sodium-blood pressure relationship [8]. In support of this, it is worth to mention results of a recent international study (INTERMAP Research Group) carried out to determine dietary potassium intake in Japanese participants aged 40–59 years. Here, potassium intake per 1000 kcal of total energy intake, with participants stratified by urinary potassium excretion per unit of body weight (UK/BW) quartiles, was significantly different (P for trend < 0.001) [7], suggesting that due to the cross-sectional nature of the study by Minamino et al. [1], and non-random enrollment of the participants in their cohort, it is quite rational to assume that dietary potassium intakes in RA outpatients in the study of Minamino et al. to be different as well. This is especially important since daily dietary potassium intake can be as large as the extracellular potassium pool [9]. This is again relevant because Minamino et al. [1] have used spot urine samples to estimate salt intake using Tanaka formula [10], which has recently been questioned, mean differences for the Tanaka formula compared to the referred 24-h urinary sodium excretion was significantly different (difference (95% CI), 542 (261, 824); P < 0.001) [11].

Secondly, NaCl cotransporter (NCC) activation, during hypovolemia, by the renin-angiotensin system stimulates sodium reabsorption and thus preventing potassium secretion. While NCC inactivation following high dietary potassium intake maximizes kaliuresis and limits sodium retention, in spite of high aldosterone levels, NCC activation following a low-potassium diet contributes to salt-sensitive hypertension [9]. It appears that due to the lack of available data on potassium intake and other confounding factors, current data analysis was not sufficient to draw a solid result.

Third, sodium depletion results in increased renin expression in different tissues such as kidney, heart, and adrenal. Renin expression is regulated in a tissue-specific manner. This highlights different functions for the sodium-responsive and -nonresponsive systems [12]. This evidence is relevant since components of renin-angiotensin system are recently shown to be associated with disease activity and subclinical atherosclerosis in rheumatoid arthritis [13] and are differentially expressed in patients with RA and osteoarthritis [14]. No evidence of sodium depletion is provided by Minamino et al. [1], and it is difficult to judge if some associations with RA disease activity and hypertension was partly or totally mediated by renin-angiotensin system.

These imply that their conclusion stating that urinary Na/K ratio is an independent indicator associated with current RA disease activity and hypertension could be either an artifact or association in some patients might be more pronounced (most probably), while in some patients, associations might be attenuated (less probably). Authors might choose to re-analyze their data, particularly if they could adjust for confounding factors such as rennin-angiotensin, in case data were available. A mediation analysis performed to support the conclusion is also recommended.

## Data Availability

Data are in the published article and reference citations.
